# The Impact of COVID-19 on Maintaining Physical Activity and Its Associated Factors among Medical Students

**DOI:** 10.3390/ijerph192315752

**Published:** 2022-11-26

**Authors:** Ahmed M. Wafi, Amani A. Mosleh, Amani A. Mutaen, Dalal F. Hakami, Rawdah A. Baeshen, Shahad A. Rajhi, Joud M. Alamri, Ahmad Y. Alqassim

**Affiliations:** 1 Physiology Department, Faculty of Medicine, Jazan University, Jazan 45142, Saudi Arabia; 2 Faculty of Medicine, Jazan University, Jazan 45142, Saudi Arabia; 3 Family and Community Medicine Department, Faculty of Medicine, Jazan University, Jazan 45142, Saudi Arabia

**Keywords:** physical activity, exercise, lifestyle medicine, COVID-19, medical students, public health

## Abstract

Background. Stay-home orders and the shutdown of university campuses and fitness centers have greatly influenced health behaviors, resulting in a widespread reduction in physical activity. This study aimed to identify factors associated with maintaining physical activity among Jazan University medical students during the COVID-19 shutdown. Methods. An official Arabic short form of the International Physical Activity Questionnaire was used to assess physical activity before and during the COVID-19 home quarantine. Differences between groups were examined using chi-square analyses and the Mann–Whitney U test. Multinomial logistic regression was used to test whether certain demographic and exercise-related variables were significant predictors of maintaining physical activity levels during the COVID-19 pandemic. Results. More than half of the participants (53%) reported no change or increased physical activity level. Several factors were significantly related to physical activity heterogeneity including income *(p* = 0.04), fitness center membership (*p* < 0.01), usage of fitness tracker devices *(p* < 0.01), and the purchase of physical activity equipment during the COVID-19 pandemic *(p* < 0.01). The odds of maintaining physical activity were higher among those who tracked their physical activity (AOR = 6.160; 95% CI = 3.782–10.032, *p* < 0.001). Similarly, the odds of maintaining physical activity were higher among those who purchased home-exercise equipment during the pandemic (AOR = 2.243; 95% CI = 1.386–3.632, *p* = 0.001). Conclusions. Understanding the factors affecting physical activity behaviors is an essential step toward addressing the reduction in physical activity. This could help maintain physical activity levels during a potential future pandemic-related shutdown.

## 1. Introduction

The coronavirus disease (COVID-19) has spread to more than 200 countries and affected more than six million people up to October 2022 [1]. The virus is highly transmissible and spreads primarily via airborne droplets when infected people gather with others in enclosed spaces [2]. In early 2020, the World Health Organization (WHO) and the Center for Disease Control and Prevention (CDC) issued statements recommending social distancing to limit exposure to the very contagious and highly transmissible virus [3,4]. Subsequently, many countries have issued stay-home orders to effectively slow the spread of the virus and prevent the health system from collapsing. Saudi Arabia was among the first countries to implement preventive measures to mitigate the impact of the pandemic. In April 2020, the Saudi government issued a 24-h curfew and enforced a lockdown that included gyms and fitness centers [5].

These restrictions substantially influenced millions of people’s daily physical activity (PA, defined as any bodily movement produced by skeletal muscles that results in energy expenditure above the basal level) in Saudi Arabia and worldwide [6,7]. Several studies reported a reduction in PA and increased sedentary behaviors because of COVID-19-related restrictions [8,9,10]. These detrimental behaviors have been linked to decreased cardiorespiratory fitness and increased risk of chronic health conditions such as cardiometabolic disease, obesity, and premature mortality [11,12,13]. More importantly, avoiding sedentary behaviors, maintaining PA, and exercising amid COVID-19 are important preventive measures to reduce adverse outcomes and improve survival [14].

Although PA levels are likely to decline during a pandemic-related shutdown, previous analyses have revealed a substantial difference in PA behavior response to the pandemic [15,16]. For instance, some individuals have experienced the aforementioned restriction-related decline in PA, while others have adopted strategies to maintain PA levels and overcome barriers to PA despite restrictions.

Lack of time or motivation and the high cost of exercise equipment and fitness center memberships are known barriers to PA [17]. These barriers are likely to be exacerbated during the shutdown associated with the COVID-19 pandemic. However, sociodemographic factors and financial resources are likely to influence many of these PA barriers [18]. For instance, those with low income may face higher challenges when maintaining PA levels in the face of the pandemic. Furthermore, several factors can help maintain regular PA (in the absence of the pandemic) including affordable and convenient PA options. With the closure of community centers and gyms, access to exercise facilities is likely to be lower, and the ability to purchase home-based exercise equipment is also limited, especially with the financial strain associated with the pandemic [17,18].

Additionally, with a complete shift to virtual classes, university students including medical students may have increased stress and strain on mental health, which may contribute to reduced motivation and energy for exercise [19]. Although medical students have better knowledge regarding healthy lifestyles including PA relative to other students [20], it is unclear whether this knowledge would affect their behaviors and influence the sustainability of PA during the pandemic. Consequently, this study aimed to examine the characteristics of medical students who reported having difficulties in maintaining their PA level during the pandemic. We examined whether certain demographic and exercise-related factors (self-monitoring of PA and purchase of home-exercise equipment) were predictors of maintaining PA. We hypothesized that lifestyle changes such as studying from home and changes in income due to COVID-19 restriction measures would influence the PA levels and that medical students with high PA levels before the pandemic would be most affected by these restriction measures and would experience a higher reduction in their PA levels. We also hypothesized that students who tracked their PA with fitness tracker devices and purchased home-based exercise equipment during the pandemic would be protected against a reduction in PA levels. 

## 2. Materials and Methods

### 2.1. Study Design, Participants, and Sample Size

An observational cross-sectional study was conducted between 3 March and 10 April 2022 in the Faculty of Medicine at Jazan University in Saudi Arabia. The target population included students who were seeking to undertake a Bachelor of Medicine, Bachelor of Surgery (MBBS). The inclusion criteria of the participants included being a medical student and willingness to take part in the data collection via an interview. Students who reported major incidents that could interfere with their PA such as major surgeries were excluded. These criteria were assessed via screening questions at the beginning of the interview.

The sample size was calculated using the Raosoft sample size calculator (Raosoft., Seattle, WA, USA, raosoft.com, accessed on 10 January 2022). We calculated that we needed 384 participants to reach a 95% confidence interval and a 5% margin of error. However, using convenient random sampling, the sample size was increased to 439 to increase the power of our study results.

### 2.2. Data Collection Instrument

In this study, the Arabic short form of the International Physical Activity Questionnaire (IPAQ) was used to assess PA and sedentary behavior. The IPAQ is a seven-item scale based on the estimated duration and level of physical activity over the last seven days with good reliability and validity [21,22]. The participants were interviewed by trained interviewers from the research team. Since the interview with the participants was conducted only once, the level of PA for both conditions (before and after COVID-19) was assessed at the same time. We added questions to the survey to extract information on the use of personal fitness tracker devices, gym memberships, and the purchase of home exercise equipment during the lockdown. The translated version of the questionnaire was then pilot-tested among 25 students. 

We computed the weekly PA level, expressed as metabolic equivalent task minutes per week (MET-min/week), based on the concept of MET, which is equivalent to the resting metabolic rate and corresponds to 3.5 mL O_2_ Kg^−1^ min^−1^ [23]. More specifically, by using the basal level of energy expenditure assigned to each type of PA (the corresponding METs were: 3.3 for walking, 4.0 for moderate intensity PA, 8.0 for vigorous intensity PA), and the total weekly energy expenditure was estimated (i.e., the sum of walking, moderate-intensity PA, and vigorous intensity PA) in MET-min/week [24,25]. Data from IPAQ can be reported as either categorical (low, moderate, and high) or continuous measures (MET-min/week) [26]. In our study, the PA level was reported as a continuous variable, which enabled us to quantify the effects of the individual profiles (sociodemographic and PA-related factors) on the change in PA levels. Since there is no established threshold for presenting MET-minutes, data were reported as the median and interquartile range (IQR). 

The participants were asked about their PA retrospectively several months after the pandemic, which resulted in a relatively long recall period. Therefore, contrary to previous studies, we did not attempt to compare the PA before and during the lockdown per se. Instead, we aimed to understand which sociodemographic and PA-related factors were more likely to be associated with maintaining PA levels during the pandemic. The participants were categorized into two groups: those with decreased PA (reported a decline of more than 10 MET-min/week) and those with unchanged or increased PA (within 10 MET-min/week or higher). 

### 2.3. Statistical Analyses

Statistical analyses were conducted using the Statistical Package for the Social Sciences (SPSS version 25). Descriptive statistics for the demographic data were presented as the median and interquartile range or frequency (%). The Kolmogorov–Smirnov test revealed that the change in MET-min/week was not normally distributed. Thus, the non-parametric Mann–Whitney U test was used to examine the differences in the MET-min/week between independent groups. 

The differences between groups were examined with chi-square statistics for the categorical variables. The variables that showed a significant association with the change in PA level (*p* < 0.05) were included in the subsequent logistic regression to identify significant predictors for maintaining the PA levels. Pearson correlation was used to examine the relationship between the baseline PA (MET-min/week before COVID-19) and the change in PA levels (change in MET-min/week). The level of significance for all analyses was set at *p* < 0.05. Figures were produced using GraphPad Prism version 9.

## 3. Results

### 3.1. Participant Characteristics

The participant characteristics are provided in Table 1. On average, participants were 21 ± 1.8-years-old and the majority (54.7%) had a normal weight, while the minority were underweight (19.4%) or overweight (17.5%), or obese (8.4%). Most participants (65.8%) resided in villages, and the majority were unmarried (90.9%) and living with family or colleagues (95.4%). Household income was relatively equally distributed across all categories except for the highest income category, where 43.7% reported a monthly income of more than SAR 15,000. The majority (82.7%) of students reported a grade point average (GPA) between 3.75 and 5.00. Most participants (75.6%) were not members of fitness centers before the COVID-19 lockdown, and 56.5% reported using fitness tracker devices. More than half (53.5%) of the participants did not purchase exercise equipment during the COVID-19 lockdown. 

### 3.2. Demographic and PA Factors 

**Figure 1:**
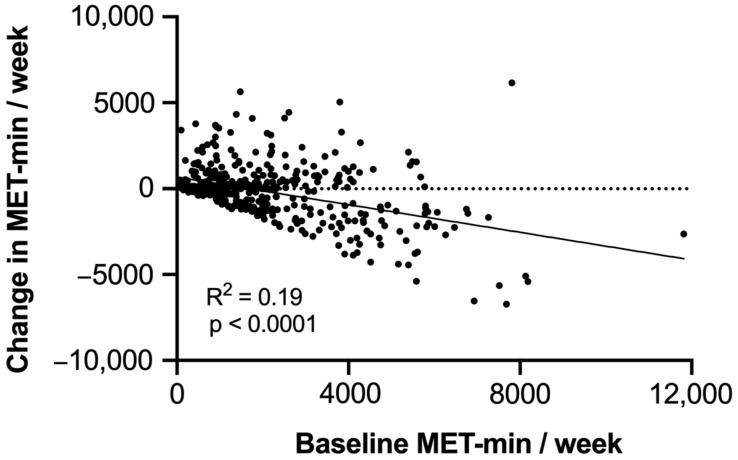
Correlation between the baseline PA levels (before lockdown) and the change in the PA levels as measured by MET-min/week.

Table 2 shows the association between the demographic factors and the change in PA level during the COVID-19 lockdown. While 205 participants (46.7%) reported a decline of more than 10 MET-min/week, 234 (53.3%) participants reported that their PA level remained relatively unchanged (within 10 MET-min/week) or even increased during the lockdown period. Chi-square analysis revealed that body mass index (*p* = 0.23) and sociodemographic factors including residence (*p* = 0.70), marital status (*p* = 0.66), GPA (*p* = 0.33), and living alone or with family (*p* = 0.44) were not significantly related to change in PA during the COVID-19 lockdown. Income (*p* = 0.04), fitness center membership before the pandemic (*p* < 0.01), usage of fitness tracker devices (*p* < 0.01), purchase of fitness equipment during the pandemic, and the baseline MET-min/week (*p* < 0.01) were significantly related to changes in PA levels during the COVID-19 lockdown. Correlation analyses revealed that there was a negative association between the baseline PA and the change in the PA levels (change in MET-min/week); students with the highest PA levels at the baseline (before COVID-19) experienced a greater reduction in their PA level (Figure 1).

**Figure 2:**
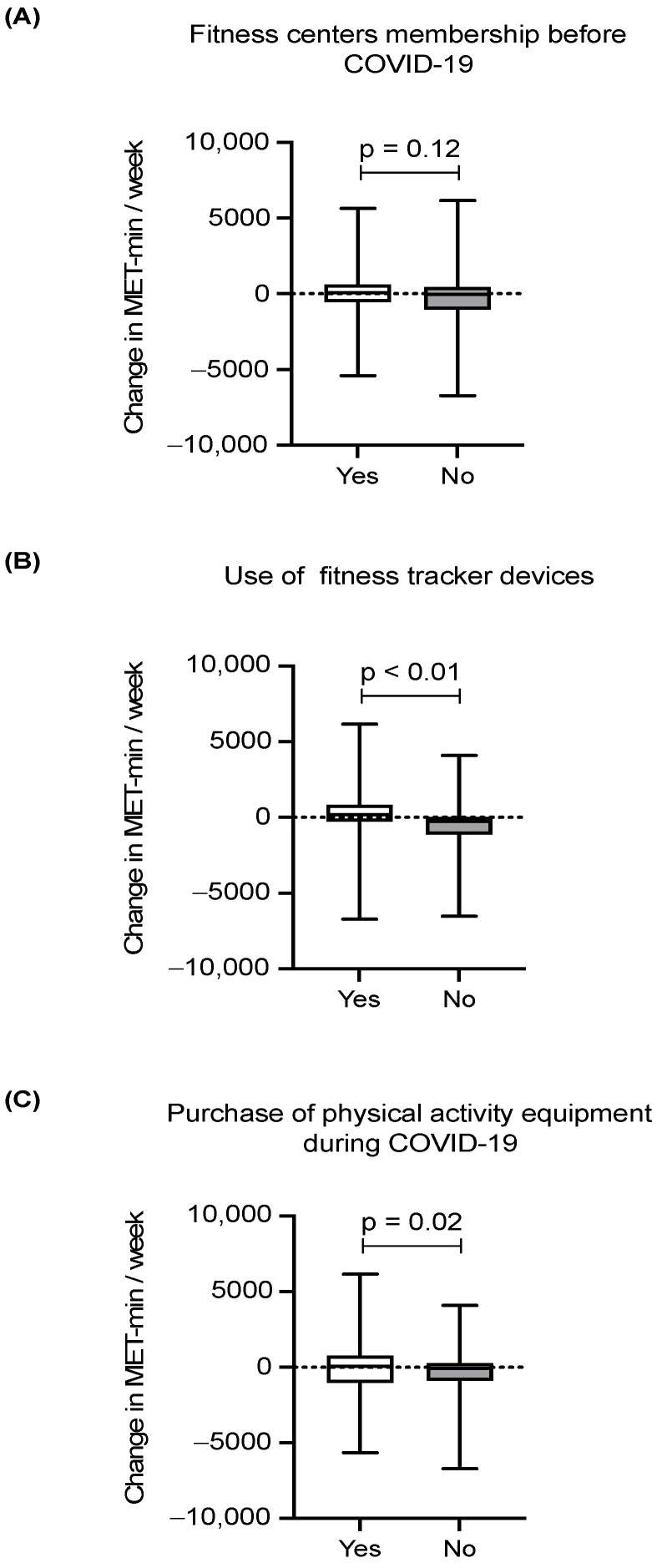
Change in PA according to fitness center membership (**A**), use of PA tracker devices (**B**), and purchase of PA equipment during the pandemic (**C**).

Figure 2 shows the median change in MET-min/week according to whether or not the participants had a gym membership before the pandemic, used fitness tracker devices, and purchased physical activity equipment during the pandemic. Those who did not use fitness tracker devices had a significantly greater reduction in PA relative to those who tracked their PA (−260 vs. 191; difference = 451 MET-min/week, *p* < 0.01). Similarly, participants who did not purchase PA equipment during the pandemic had a significantly higher PA reduction than those who did purchase PA equipment (−70 vs. 80.5; difference = 150.5 MET-min/week, *p* = 0.02). However, fitness center membership before the pandemic did not influence the change in PA level (74 vs. −40; difference = 114 MET-min/week, *p* = 0.12).

Table 3 shows the results of the multinomial logistic regression. Those who tracked their PA with tracker devices were 6.160 times more likely to maintain/increase their PA relative to those who did not track their PA (adjusted odd ratio (AOR) = 6.160; 95% confidence interval (CI) = 3.782–10.032; *p* < 0.001). Participants who purchased PA equipment during the COVID-19 pandemic were 2.243 times more likely to maintain or increase their PA level compared with those who did not.

## 4. Discussion

Previous studies have compared the PA levels before and after the COVID-19 lockdown, which indicated, for the vast majority, a reduction in PA along with an increase in sedentary behavior [27]. Other studies have shown that these trends have a deleterious effect on individual health including immune dysfunction and deterioration in mental health [28]. As a result, it is extremely important for those with normal health or chronic disease to maintain their PA to prevent or mitigate future health problems and a subsequent decline in their quality of life [29,30]. 

In this context, we aimed to identify the demographic and pre-pandemic exercise-related characteristics and strategies that helped medical students to maintain PA levels during the lockdown associated with the COVID-19 pandemic. We hypothesized that those with high PA levels before COVID-19 would experience the sharpest decline during the lockdown. Furthermore, we hypothesized that students who tracked their PA or purchased exercise equipment would be protected against a decline in PA during the pandemic. Our hypothesis was largely supported as both of the aforementioned factors seemed to mitigate the reduction in the PA. Knowledge about such factors may be important to consider when developing strategies to maintain or promote PA levels, especially during pandemics. For instance, knowledge about these factors allows for the identification and quick targeting (from the start of the pandemic) of populations including students in need of support and tools (digital technologies tracking PA and other home-based exercise equipment) that support remote PA. This, in turn, could be useful to reduce stress and increase well-being and quality of life, which may help to cope with potential future pandemics [31]. 

Our study indicates that the PA level before the pandemic was a significant predictor of maintaining PA during the pandemic. Indeed, previous studies have shown that PA levels before COVID-19 restrictions seem to influence the sustainability of the PA practice [32,33,34]. According to our study, 53.3% of medical students who reported that their PA levels remained relatively unchanged or even increased during the pandemic had lower baseline (before COVID-19) PA levels relative to those who experienced a reduction in PA during the pandemic, suggesting that the PA level at the baseline may influence PA sustainability during lockdown associated with COVID-19. While the closure of sports facilities may contribute to these findings, it is possible that the nature of PA practiced before the pandemic played a role in the sustainability of PA. For instance, those engaged in PA that was difficult to substitute and highly dependent on a specific infrastructure such as swimming may have faced more difficulties sustaining their PA [35]. However, it is important to note that medical students remained physically active during the pandemic (i.e., met the recommended PA levels [36]), with a median of 1367 MET-min/week. However, having a PA level within the recommended PA guidelines during COVID-19 was not surprising [37,38]. 

Previous studies have suggested that the absence of a gym environment and the lack of partners were essential factors for reduced exercise motivation [39,40]. Connecting with others has been associated with persistence, motivation, and physical and psychological well-being [41,42]. The absence of the positive behaviors that people were used to experiencing in the gym environment is probably one of the reasons for the lack of motivation to exercise at home and the reduction in their PA levels. However, our analysis revealed that those who had fitness center memberships before the pandemic did not experience a significantly greater reduction in their PA. In fact, those with no previous membership had a significantly greater reduction in their PA relative to the baseline PA levels. We speculate that during the latter part of the home-quarantine period and passive wait for things to return to normal, participants started to look for alternatives to exercise at fitness centers. Some of these alternatives may have included purchasing equipment to work out at home, which may have contributed to maintaining their PA. Indeed, our analysis revealed that more than half (62%) of the participants with previous fitness center memberships purchased exercise equipment during the pandemic. However, the total number of participants with previous fitness center memberships was only 107, which may limit our conclusion in this regard.

Income has been shown to influence PA, as low-income people are less likely to meet the PA guidelines than higher-income people [43,44]. In Saudi Arabia, low income is associated with lower PA levels among college students [45,46]. Those with low income were less likely to purchase PA equipment during the pandemic. However, our analysis did not show a relationship between income and the purchase of PA equipment. This finding may be explained in part by the difficulty of defining income among young college students who may be dependent on their parent’s income. Despite the well-documented economic burden of COVID-19 on many countries including Saudi Arabia, we saw a relatively high rate (46.5%) of purchasing exercise equipment during the pandemic. As young adults, students should exercise for at least 150 min of moderate-intensity or 75 min of vigorous-intensity exercise [47]. Our sample of medical students may have been informed and educated on these guidelines, which may have helped them to prioritize PA via purchasing exercise equipment during the pandemic. 

Our results are consistent with the current literature indicating that those who used fitness tracker devices and other digital technologies seem more physically active than non-users during the pandemic [48,49,50]. We saw that using fitness tracker devices seemed to protect against a decline in PA during the pandemic; this may be due to regular feedback on behavior and the ability to detect change. Nevertheless, sampling may influence this observation, which involved only medical students who may be health-conscious and tech-adopting individuals.

We acknowledge several limitations. Although interviews were made to collect data from the participants, the instrument for data collection was a questionnaire, which is vulnerable to recall bias. The study’s cross-sectional design limited our ability to make causal effect relationships. The timing of our study did not allow us to obtain a pre-pandemic response in real-time. Furthermore, convenient random sampling limited our ability to generalize the results and is vulnerable to selection bias. 

## 5. Conclusions

While students were encouraged to exercise to maintain their mental and physical health, the lockdown markedly reduced opportunities to engage in various exercise opportunities. This seemed to result in a heterogeneous effect depending on the student’s PA levels before the pandemic and other PA-related factors such as PA tracking and purchasing home-based exercise devices.

Attenuation of PA reduction, especially in the face of a future pandemic, should be a public health priority. The first step to addressing such a problem starts with understanding how sociodemographic and PA factors affect PA during pandemics, considering these factors in response actions, and developing targeted interventions in the face of a potential future pandemic. Future studies are warranted to look into the possible long-term effect of a health crisis on PA by means of longitudinal study designs and measuring PA in an objective manner before, during, and after other health crisis-related restrictions.

## Figures and Tables

**Table 1:** The sociodemographic structures of the study participants.

Characteristics	*n*	%
Gender		
Male	179	40.8
Female	260	59.2
BMI		
Underweight	85	19.4
Normal weight	240	54.7
Overweight	77	17.5
Obese	37	8.4
Residence		
City	150	34.2
Village	289	65.8
Marital status		
Married	40	9.1
Unmarried	399	90.9
Living with		
Alone	20	4.6
With family or colleagues	419	95.4
Household income (SAR/month)		
<5000	94	21.4
5000–9999	79	18.0
1000–14,999	74	16.9
>15,000	192	43.7
Grade point average (GPA)		
<2.00	2	0.5
2.00–2.74	9	2.1
2.75–3.74	65	14.8
3.75–4.49	137	31.2
4.50–5.00	226	51.5
Were you a member of a fitness center before the COVID-19 pandemic?		
No	332	75.6
Yes	107	24.4
Usage of fitness tracker devices		
No	191	43.5
Yes	248	56.5
Purchase of home-based exercise equipment during the COVID-19 pandemic		
No	235	53.5
Yes	204	46.5

BMI: Body mass index; SAR: Saudi Riyal (SAR 1 = USD 0.27).

**Table 2:** The sociodemographic structures of the study participants according to the change in PA level during the COVID-19 pandemic.

Characteristics		No Change or Increase in PA *n* = 234 (53.3)		Decrease in PA *n* = 205 (46.7)		*p*-Value
		*n*	%	*n*	%	
Gender	Male	93	52	86	48	0.63
	Female	141	54.2	119	45.8	
BMI	Underweight	41	48.2	44	51.8	0.23
	Healthy weight	130	54.2	110	45.8	
	Overweight	47	61.0	30	39.0	
	Obese	16	43.2	21	56.8	
Residence	City	78	52.0	72	48.0	0.70
	Village	156	54.0	133	46.0	
Marital status	Unmarried	214	53.6	185	46.4	0.66
	Married	20	50.0	20	50.0	
Living with	Alone	9	45.0	11	55.0	0.44
	Family	225	53.7	194	46.3	
Household income (SAR/month)	<5000	43	45.7	51	54.3	0.04
	5000–9999	37	46.8	42	53.2	
	10.000–14,999	37	50.0	37	50.0	
	>15,000	117	60.9	75	39.1	
Grade Point Average (GPA)	<2.00	2	100.0	0	0.0	0.33
	2.00–2.74	9	44.4	5	55.6	
	2.75–3.74	33	50.8	32	49.2	
	3.75–4.49	73	53.3	64	46.7	
	4.50–5.00	122	54.0	104	46.0	
Fitness centers membership before COVID-19	Yes	72	67.3	35	32.7	<0.01
	No	162	48.8	170	51.2	
Use of fitness tracker devices	Yes	171	69.0	77	31.0	<0.01
	No	63	33.0	128	67.0	
Purchase of fitness equipment during COVID-19	Yes	124	60.8	80	39.2	<0.01
	No	110	46.8	125	53.2	
Total MET-minutes per week before COVID-19, median (IQR)		1081.5 (1610)		1980 (2857)		<0.01
Sitting time before COVID-19, median; IQR		540 (495)		480 (420)		0.36

We used the chi-square test for categorical variables and the Mann–Whitney U test for continuous variables. BMI: Body mass index; SAR: Saudi Riyal (SAR 1 = USD 0.27).

**Table 3:** The results of multinomial logistic regression predicting maintaining of the PA level during the pandemic.

Variable		AOR (95% CI)	*p*-Value
MET-min/week before COVID-19		0.999 (0.999–1.000)	<0.001
Household income (SAR/month)	<5000	0.623 (0.348–1.114)	0.111
	5000–9999	0.409 (0.215–0.779)	0.006
	10,000–14,999	0.666 (0.354–1.251)	0.206
	>15,000	Ref.	
Fitness center membership before COVID-19	Yes	1.427 (0.825–2.467)	0.203
	No	Ref.	
Use of fitness tracker devices	Yes	6.160 (3.782–10.032)	<0.001
	No	Ref.	
Purchase of physical fitness equipment	Yes	2.243 (1.386–3.632)	0.001
	No	Ref.	

AOR: adjusted odd ratio; CI: confidence interval; Ref: reference category for the categorical data.

## Data Availability

The datasets generated and analyzed during the current study are available from the corresponding author upon reasonable request.
